# Dynamix: dynamic visualization by automatic selection of informative tracks from hundreds of genomic datasets

**DOI:** 10.1093/bioinformatics/btx141

**Published:** 2017-03-11

**Authors:** Matthias Monfort, Eileen E M Furlong, Charles Girardot

**Affiliations:** European Molecular Biology Laboratory, Genome Biology Unit, Heidelberg, Germany

## Abstract

**Motivation:**

Visualization of genomic data is fundamental for gaining insights into genome function. Yet, co-visualization of a large number of datasets remains a challenge in all popular genome browsers and the development of new visualization methods is needed to improve the usability and user experience of genome browsers.

**Results:**

We present Dynamix, a JBrowse plugin that enables the parallel inspection of hundreds of genomic datasets. Dynamix takes advantage of *a priori* knowledge to automatically display data tracks with signal within a genomic region of interest. As the user navigates through the genome, Dynamix automatically updates data tracks and limits all manual operations otherwise needed to adjust the data visible on screen. Dynamix also introduces a new carousel view that optimizes screen utilization by enabling users to independently scroll through groups of tracks.

**Availability and Implementation:**

Dynamix is hosted at http://furlonglab.embl.de/Dynamix.

**Supplementary information:**

Supplementary data are available at *Bioinformatics* online.

## 1 Introduction

The significant drop in sequencing costs made large genome-scale studies like ENCODE (Consortium, 2012) a reality. Such projects have resulted in thousands of publicly available genomics datasets that represent an invaluable source for new discoveries. To this end, human visual inspection of the data remains essential as new hypotheses often result from a meticulous exploration of newly acquired data and its comparison with established datasets. In popular genome browsers (Hung and Weng, 2016; Stein, 2013; Thorvaldsdottir *et al.*, 2013; Westesson *et al.*, 2013; Yates *et al.*, 2016), datsets are visualized as tracks where a track represents both a dataset and its visualization options. Visualized tracks are aligned to a common reference sequence and stacked on top of each other (in the *track container*) to allow for direct comparison (Supplementary Fig. S1). Users typically interact with the data by zooming in and out, panning left and right or using embedded search features (by genomic feature names or location). Although thousands of datasets are publicly available, it remains challenging to visualize more than a couple of dozens tracks in current web genome browsers. Indeed, stacking too many tracks usually comes at the cost of smooth navigation due to the time needed to render the data and the limited size of the screen (which forces the user to constantly scroll up and down and re-organize the tracks). JBrowse (Westesson *et al.*, 2013) has brought significant improvements to the former point through, for instance, the implementation of client-side rendering, query-efficient data structures and smooth—«Google Maps like»—navigation system. Desktop applications like IGV (Thorvaldsdottir *et al.*, 2013) also eliminate the downside of shared-server resources using the user machine resources. However, the limitations related to the user’s hardware (RAM, CPU) and the screen size remains and severely limit the number of tracks that can be displayed. While a set of tracks may be relevant to display at a given genomic location, the same tracks may no longer be relevant to display a few hundred base pairs downstream where the signal drops to background level. As the navigation continues, the user must scroll up and down, swap tracks in the track container and drag tracks next to each other. To circumvent these issues, we present Dynamix, a JBrowse plugin that takes advantage of a priori knowledge to automatically display relevant tracks as the user browses the genome and offers new innovative ways to browse tracks organized in logical groups.

## 2 Results

### 2.1 Dynamic track display

**Fig. 1:**
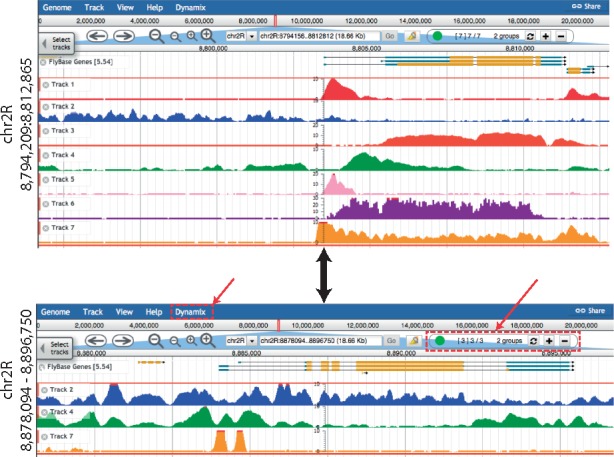
Dynamic update of the track container. Dynamix-enabled JBrowse configuration showing (from top to bottom) Flybase genes followed by 7 tracks managed by Dynamix altogether defined as a group. **Top**, screenshot for the genomic range 8 794 209–8 812 865 of chr2R where all 7 Dynamix managed tracks are displayed. **Bottom**, screenshot showing the same configuration after the user moved 84 Kb away to the genomic range 8 878 094–8 896 750 of chr2R. Dynamix still displays the Flybase genes (not managed by Dynamix) while only 3 of the original 7 tracks managed by Dynamix (Track 2, 4 and 7) remain visible; the four missing tracks have been automatically hidden from the track container by Dynamix. The visible track group (i.e. Track 1 to 7 in A) was configured to be anchored below the ‘Flybase Genes’ track. Red dashed boxes (pointed by red arrows) indicate the Dynamix specific menu and control widget

A key concept behind Dynamix is to anticipate users’ actions and execute them automatically. Practically, for a given genomic window Dynamix removes tracks without features or containing only background level signal (*locus-specific empty* tracks) from the track container and replaces them with tracks containing features or signal above background (*locus-specific interesting* tracks). Quantitative signal datasets (e.g. 4C-seq or ChIP-seq coverage) are usually associated with feature datasets (e.g. peak lists) that describe the regions containing signal. This is obviously true for published datasets but also for in-house data once data processing has taken place. In Dynamix, a quantitative signal dataset is always registered together with its companion feature dataset, which describes the interesting regions with significant signal. Upon registration, Dynamix uses both datasets to create standard JBrowse tracks and remembers them as a dependent track set. A special *dynamix_features* track compiles all features from all track sets registered in Dynamix. As the user interacts with the browser, the *dynamix_features* track is queried to assemble the list of locus-specific interesting tracks and update JBrowse’s track container (i.e. missing interesting tracks are added following a pre-defined order while empty locus-specific tracks are removed, Fig. 1 and Supplementary Fig. S2). In addition, summary information is communicated to the user through the Dynamix widget (Fig. 1, Supplementary Figs S2, S3A). Dynamic track management only occurs for tracks registered in Dynamix and it is always possible to manually add *static* tracks (JBrowse tracks not registered in Dynamix) in the track container, as with standard viewers.

To provide more flexibility, and complement Dynamix’ automated track updating, Dynamix also offers a *manual* mode that switches off the automatic updating of the track container. This results in genome browsing with static track selection (as conventional browser), but with a Dynamix widget that keeps the user informed (Supplementary Fig. S3A). In manual mode, explicit user interaction with the Dynamix widget is required to update the track container (Supplementary Fig. S3A).

### 2.2 Track groups and carousel browsing

Juxtaposing related datasets in the browser is very common to better compare the data at hand, and some genome browsers offer defining track groups (Hung and Weng, 2016; Stein, 2013; Thorvaldsdottir *et al.*, 2013; Yates *et al.*, 2016). Dynamix brings this feature to JBrowse allowing managed tracks to be organized into different *track groups*. Each track group has its own configuration (Supplementary Fig. S3A) and its browsing mode can be individually set to automatic, manual or disabled (disabled mode instructs Dynamix to simply ignore the track group).

Controlling where tracks are automatically added into the track container is extremely important to achieve a consistent and reproducible user experience and Dynamix obeys different rules to achieve this goal. First, the tracks of a track group are always rendered in a continuous block (static tracks and tracks from a Dynamix track group remain separate). Second, the display order of tracks within a group is pre-defined and fully customizable. Finally, track group anchoring within the track container can be configured such that a track group is always displayed right below a particular static track or track group (Fig. 1, Supplementary Fig. S2A).

At particular genomic locations, the number of interesting tracks can be large and exceed the user’s hardware capabilities, possibly resulting in a browser crash. To address this issue, the maximum number of tracks to display can be configured both globally (to match user’s hardware capabilities) and for each track group (to fine-tune user’s screen usage, Supplementary Fig. S3B). When the number of interesting tracks exceeds the configured maxima, only a fraction of the interesting tracks is rendered and a visual cue warns the user that data is missing from the display (Supplementary Figs S3A and S4A). While this simple solution prevents the browser from crashing, the user has no chance to view the hidden interesting tracks. In Dynamix, we solve this issue by introducing carousel browsing which allows infinite scrolling through all interesting tracks of a group. Upon each click on the carousel control, half of the visible interesting tracks are replaced with hidden tracks following the predefined track order (Supplementary Fig. S4).

### 2.3 Comparison with other genome browsers

Organizing tracks into broad categories (e.g. Genes, Variation, Repeats) is a common feature of Ensembl, UCSC and GBrowse genome browsers. JBrowse natively offers a similar (yet more general) solution based on track filtering using user-defined track annotations. In GBrowse, related subtracks (e.g. RNA-seq time series) can be grouped into a single track, subtracks can then be individually toggled in the track and their display order controlled. In contrast, Dynamix track grouping also offers control over the display order of the tracks; in addition, it provides the carousel view and automated selection of informative tracks instead of manual filtering of the subtracks. Dynamic browsing and carousel browsing are, to the best of our knowledge, unique to Dynamix.

## 3 Conclusion

Dynamix enables scientists to browse hundreds of datasets using new visualization concepts. Readers can try Dynamix using the 4CBrowser (http://furlonglab.embl.de/4CBrowser) and the DynamixDemo server which demonstrates how Dynamix can be used for rich displays (http://furlonglab.embl.de/DynamixDemo). Visualization of genomics datasets remains a challenging area of research, we hope that Dynamix will encourage the development of innovative visualization methods.

## Supplementary Material

Supplementary Data
