# Supplementary material for: Dynamix: dynamic visualization by automatic selection of informative tracks from hundreds of genomic datasets
Source: Bioinformatics. 2017 Mar 11;33(14):2194–6. doi: 10.1093/bioinformatics/btx141 (PMC5870560; doi:10.1093/bioinformatics/btx141)
Supplement: Supplementary Data [file btx141_supp.docx]

**Dynamix: Dynamic visualization by automatic selection of informative tracks from hundreds of genomic datasets.**

Matthias Monfort, Eileen E.M. Furlong and Charles Girardot

**Supplementary Figures**

**Supplementary Figure 1**


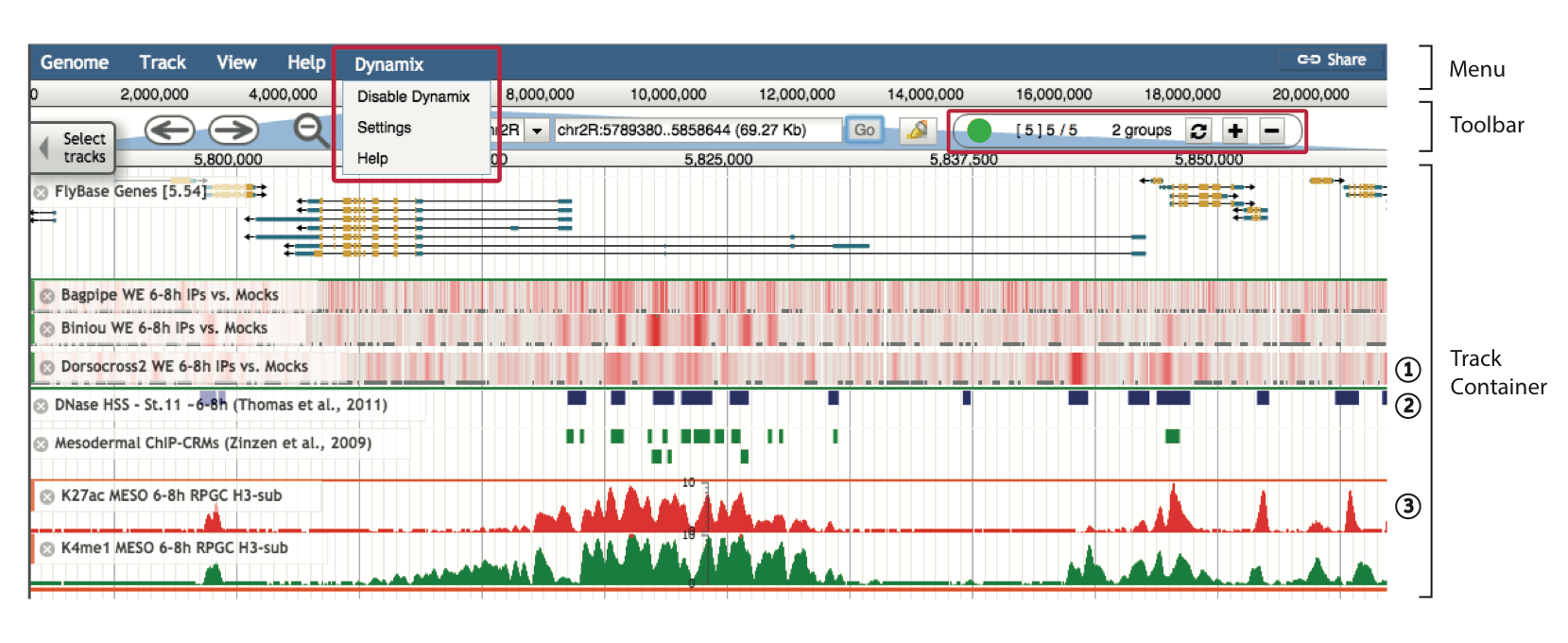


**Supplementary Figure 1. Overview of the JBrowse user interface with Dynamix plugin enabled.** Top, JBrowse traditional menu bar augmented with the specific Dynamix-specific menu (boxed in red) from which users can switch off Dynamix (“Disable Dynamix”), configure Dynamix (“Settings”) and find help (“Help”). Below the Menu bar, the Dynamix main widget (boxed in red) is embedded in the JBrowse Toolbar. Finally, tracks of data are visible in the track container and displayed according to the tracks configuration. Quantitative signal tracks can be displayed as heat maps (e.g. the “Dorsocross2 WE 6-8h IPs vs. Mocks” track marked “1”) or XY plots (e.g. the “K27ac MESO 6-8h” track marked “3”) while features are traditionally displayed as coloured rectangles (e.g. the “Mesodermal ChIP-CRMs (Zinzen et al., 2009)” track marked “2”).

**Supplementary Figure 2**

**Supplementary Figure 2. Dynamic update of the track container.** Dynamix-enabled JBrowse configuration showing (from top to bottom) Flybase genes followed by 7 tracks managed by Dynamix altogether defined as a group. A, screenshot for the genomic range 8,794,209 to 8,812,865 of chr2R where all 7 Dynamix managed tracks are displayed. B, screenshot showing the same configuration after the user moved to the genomic range 8,878,094 to 8,896,750 of chr2R. Dynamix still displays the Flybase genes (not managed by Dynamix) while only 3 of the original 7 tracks managed by Dynamix (Track 2, 4 and 7) remain visible; the four missing tracks have been automatically hidden from the track container by Dynamix. The visible track group (i.e. Track 1 to 7 in A) was configured to be anchored below the “Flybase Genes” track. C & D, the hidden “dynamix_features” track compiles all features from all track sets registered into Dynamix. After each user interaction (panning and zooming the visualization window or change of location by gene name query), Dynamix queries the “dynamix_features” track and assembles the list of interesting tracks from the names of matched features. C, content of the “dynamix_features” track for the genomic range visible in (A). D, content of the “dynamix_features” track for the genomic range visible in (B).

**Supplementary Figure 3**

**
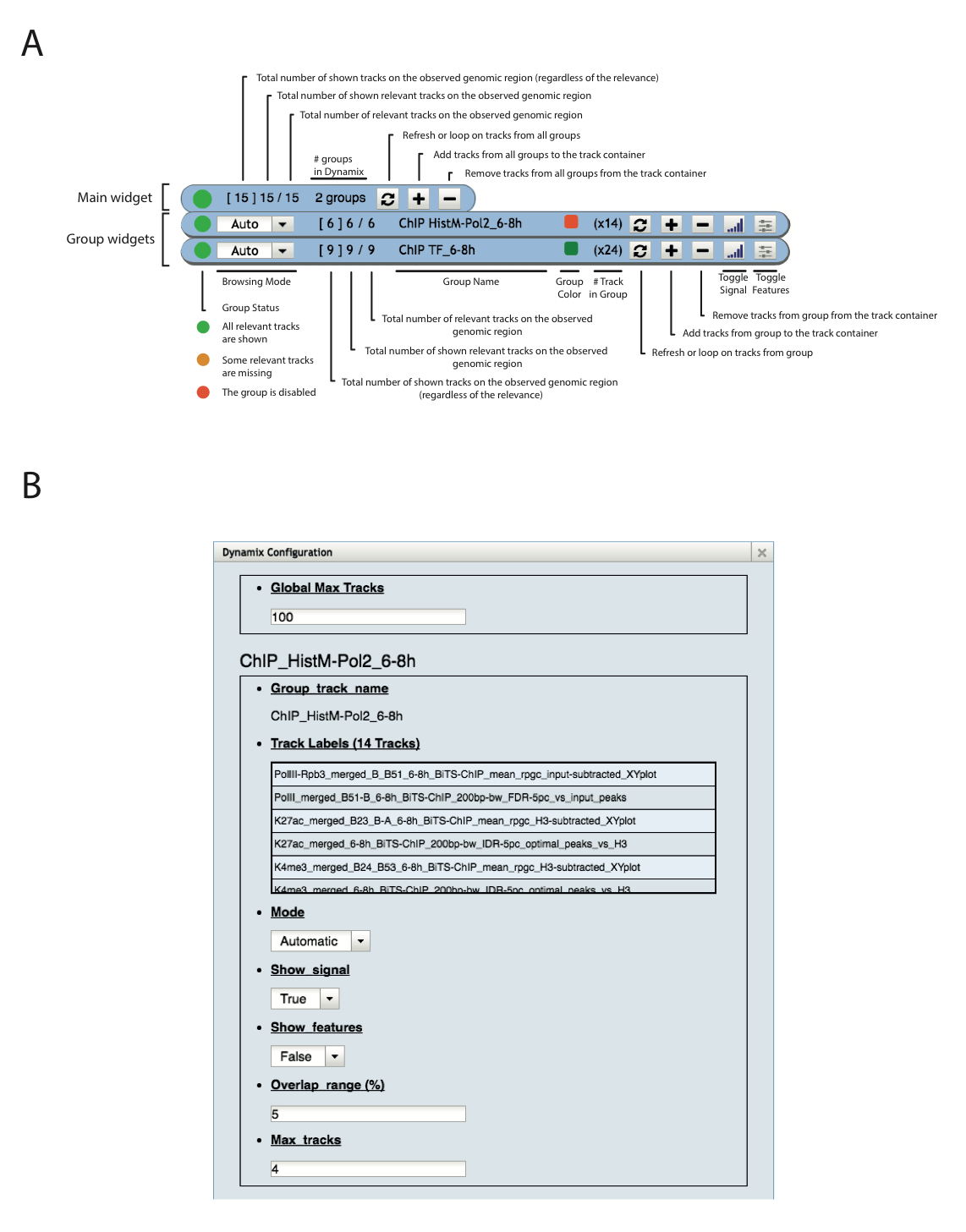
**

**Supplementary Figure 3. Details of the Dynamix user interface.** A, the main Dynamix widget (top) and 2 individual group widgets (bottom). In manual mode, explicit user interaction with the Dynamix widget is required to update the track container: the user can either add missing tracks (while obsolete locus-specific tracks are not removed) or completely refresh the display i.e. mimicking the Dynamix automatic mode. When multiple groups are defined, the information displayed through the master widget is a summary of the individual group information, and individual track group statistics and settings (browsing mode and relevant controls specific to the manual mode) can be readily accessed by clicking on the master widget. B, details of the Dynamix “Settings” menu showing the “Global Max Tracks” parameter (the overall maximum number of tracks Dynamix is allowed to add in the track container i.e. this number does not include the static tracks that might be present in the track container) and the configuration for a track group named “ChIP_HistM-Pol2_6-8h”. The track group configuration includes (from top to bootom): the list of all track sets registered in the group, the browsing mode (one of automatic, manual or disable), the visibility (True/False) of the signal and feature tracks of the track sets, the overlap range (additional size to consider outside the visible window to fetch interesting tracks, expressed in percentage of the current window) and the maximum number of tracks Dynamix can display for this group.

**Supplementary Figure 4**


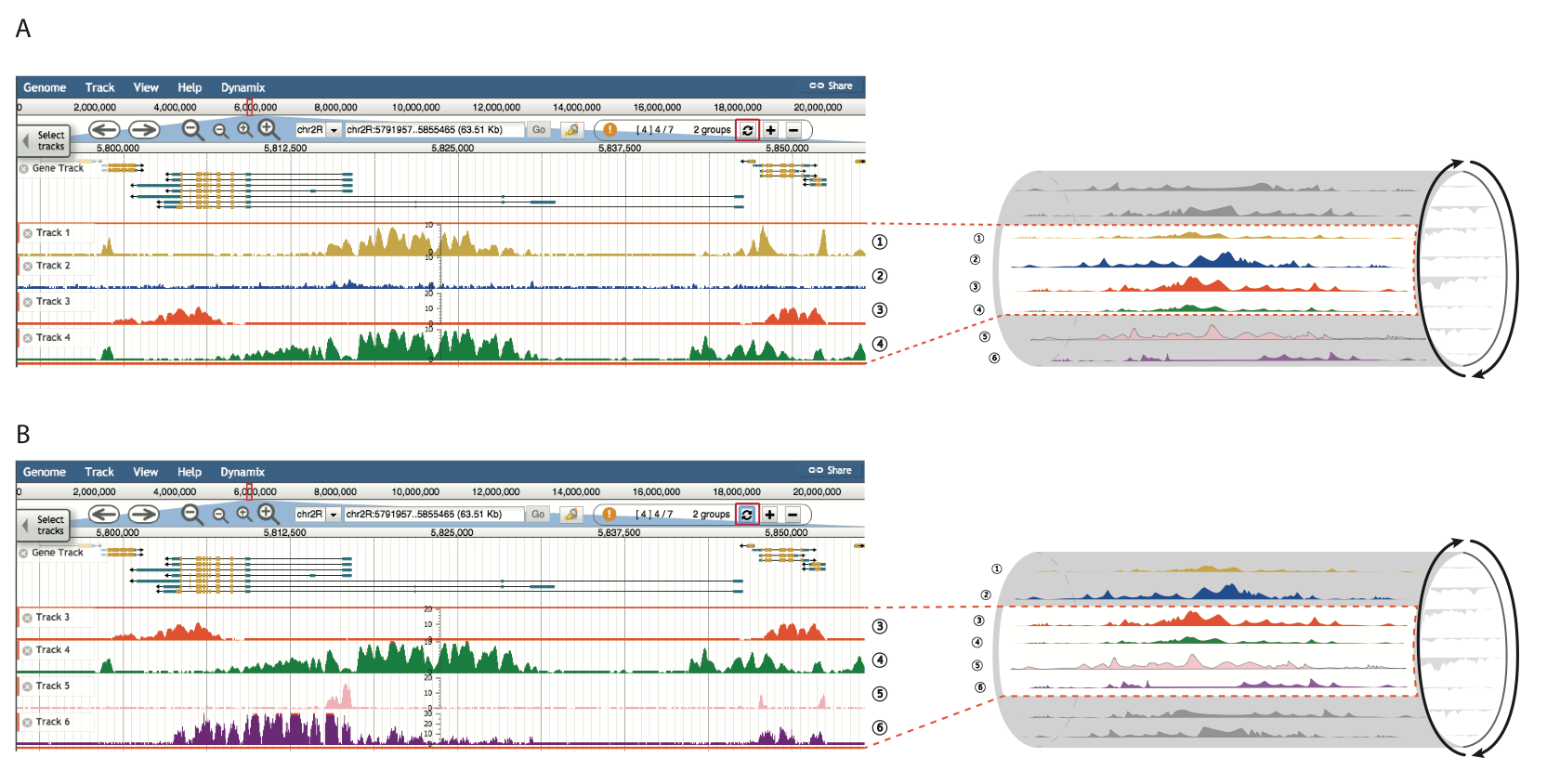


**Supplementary Figure 4. Carousel Browsing.** A left, screenshot of a region where the maximum number of tracks allowed to display has been reached (main widget displays an orange visual warning and details that only 4 of 7 interesting tracks are displayed). When interesting tracks are missing from display, the Refresh button of the Dynamix Main widget (boxed in red) becomes the carousel controller. Refresh/carousel controllers for individual track groups are available in the individual group widget (Suppl. Fig. 3A). A right, schematic of the carousel track browsing concept with 4 visible tracks (numbered 1 to 4) as shown on the left screenshot. Each click on the carousel controller rotates the carousel clock-wise. B, same region as in A after the user clicked once on the carousel controller. Half of tracks have been replaced with hidden tracks (tracks 3 to 6 are now visible). The carousel can loop indefinitely.
